# Adolescent Sex and Psyche in Brazil: Surveillance, Critique and Global Mental Health

**DOI:** 10.1007/s11013-019-09659-0

**Published:** 2019-11-15

**Authors:** Dominique P. Béhague

**Affiliations:** 1 Center for Medicine, Health and Society, Vanderbilt University, Nashville, USA; 2 Department of Global Health & Social Medicine, King’s College London, London, UK

**Keywords:** Global mental health, Critique, Culture, History, Research technologies, Adolescence, Teen pregnancy

## Abstract

Drawing on a historical ethnography conducted in Southern Brazil, this article explores how public health programs for adolescent reproductive and mental health have emerged in Brazil and begun to intersect with the growing field of “global mental health” (GMH). The story I recount begins not in the 2010s with the rapid rise of expert interest in adolescent health within GMH, but in the 1990s, the decade when young teens in Brazil were first coming into contact with practices and approaches in research, schools and clinics that have both underpinned *and* critiqued the production of an adolescent mental and reproductive health sub-field. In parsing what young women’s encounters with the then newly-emerging questionnaires, measurement tools, school-based programs and clinical practices came to mean to them, I use a genealogical approach to consider how histories of education reform, population control, psychoanalysis, social medicine, the transition to democracy, feminism and grass-roots politics all entered the fold, shaping the way adolescent sex-and-psyche materialized as a contested object of expertise. I end by exploring what this case can teach global mental health advocates and social theorists about practices of critique.

## Introduction

In 2011, a seminal article on the mental health of children and adolescents appeared in the second *Lancet Series* calling for more attention to global mental health (GMH), the first series having been published in 2007 (Horton 2007; Kieling et al. 2011). Endorsed by the World Health Organization, GMH aims to reduce stigma, ensure the protection of people with mental disorders, and increase access to treatment for people with mental disorders in low- and middle-income countries. In the past decade, GMH researchers have successfully broadened the scope of their work by emphasizing links between mental and physical health and forging alliances with reproductive health, non-communicable diseases and, most recently, child and adolescent health (Patel and Chatterji 2015). The power of these alliances rests on underscoring “comorbidities” between psychiatric problems such as attention-deficit disorder and conduct problems, and other outcomes such as substance abuse, violence and sexual “risk behaviors” (Patel et al. 2007). Concurrent trends in psychiatric epidemiology have progressively reframed teen sexuality and pregnancy as a problem of the developing psyche, at times shifting policy attention away from the role of poverty and access to education in shaping adolescent reproductive life (Chalem et al. 2012). Adolescent health and risk behaviors are now routinely seen as key predictors of psychiatric and physical health problems in adults, from severe depression, bipolar disorder and psychosis to diabetes and cancer, making young people’s mental and sexual health pivotal for the future health of whole populations (Sawyer et al. 2012).

The rise of GMH has generated considerable controversy among social scientists and transcultural psychiatrists (Whitley 2015).^1^ GMH policy, some claim, has fallen largely in line with biomedical and pharmaceutical forms of psychiatry that have become normative in countries such as the US and UK since the 1980s. Drawing from anti-psychiatry epistemologies, critics argue that this form of psychiatry has facilitated the unprecedented exportation of interventions that favor “Westernized” neuroscience models of the mind and targeted cost-effectiveness models of treatment impact, leading to forms of medicalization that ignore the social, political and economic forces accounting for ill-health and that fail to recognize the benefits of local culturally-relevant therapeutics (Mills and China 2016; Summerfield 2012). Many are also concerned that early intervention risks unduly pathologizing healthy children by focusing on behaviors that are either largely institutionally produced (such as behavioral problems in schools), developmentally “normal” (such as experimenting with drinking) or locally sanctioned in some contexts (such as young motherhood) (Skovdal 2012).

In light of these critiques, GMH leaders have made efforts to distance the subfield from the mired debates that have long vexed psychiatry. Recent GMH policies have also explicitly mobilized the culture concept by calling for the inclusion of local taxonomies of illness in research and therapeutic provisions, sometimes initiating collaborations with traditional healers (Kohrt et al. 2013). An editorial written by two prominent GMH leaders unleashed a harsh critique of the rapid proliferation of diagnostic categories put forth in psychiatric diagnostic manuals, calling for interventions that move beyond medication and talk-therapy and that put people’s preferences at the center (Jacob and Patel 2014). And in a recent GMH textbook, a chapter devoted to the history of the field attributes recent innovations in the field not to psychiatry but to the work of non-governmental organizations and civil society (Cohen et al. 2014). In the context of the repeated critique that GMH is but an arm of Western psychiatry, this text can be read as an attempt to delineate a fresh start for the field.

Fresh starts are, however, hard to come by in settings where psychiatric and medical institutions have long been embroiled in colonial and neo-colonial enterprises and their enduring forms of power (Costa 1976). Stefan Ecks has astutely argued that GMH was “formed when disparate fields and concerns were assembled in a new way” and thus should be thought of as “one moment in a long series of globalization processes in psychiatry” (Ecks 2016:804–805). Drawing from anthropological works on “global assemblages,” we might consider how GMH takes shape within techno-constellations that render the field’s policies and research priorities highly mobile. Yet as Stephen Collier and Aiwa Ong argue, assemblages do not follow given mappings; rather, they create sites in which particular forms of living and expert practices become subject to intense ethical and political reflection (Collier and Ong 2005). Further, Anne Lovell has instructively called for more expansive theorization of how different biopolitical assemblages “affect one another, even co-exist” in ways that shape not only “the management of the clinical object in question, but [also] associated practices of social inclusion, identity making, and citizenship” (Lovell 2013:128 and 132). As Paul Rabinow has written, assemblages are dependent upon, but also distinct from, larger problematizations for they entail, to use his term, “effervescent experiments” (Rabinow 2003:56). My reading of this effervescence includes the ways that “critique” becomes constitutive of how assemblages gather force and mobility.

In Brazil, collaborations with GMH experts are taking shape in the context of considerable epistemic heterogeneity and long-standing tensions in the history of Brazilian psychiatry (Ortega and Wenceslau 2015). On the one hand, biomedical perspectives in psychiatry, and in relation to adolescent mental health specifically, have grown significantly over the past few years, in part through new international partnerships.^2^ On the other hand, until recently, GMH proper has been largely absent in Brazil both because of the geopolitics of how research is funded and led by institutions in the so called “global North” and because significant segments of Brazilian psychiatry have been epistemically out of step with dominant trends in GMH.^3^ Much like in US and UK, psychoanalysis rose to prominence in the first half of the twentieth century, but when the Brazilian government initiated psychiatric reform after the end of Brazil’s 20 year-long dictatorship (1964–1984), biomedical psychiatry did not fully marginalize psychoanalysis or psychosocial perspectives, as was the case in the US and UK (Amarante 1994). Rather, reform opened a space for psychoanalytic theories to be critiqued, modified and hybridized with Brazil’s long-standing schools of social medicine and critical pedagogy (Russo 2014). Thus, critical perspectives have remained pervasive in everyday practice, with prominent Brazilians in psychiatry, education and public health working to avoid the kind of bio-medicalization that has become a defining feature of psychiatric practice in the US and UK (Tenorio 2002).

In this paper, I trace the incipient rise of expert interest in adolescent sexual, reproductive and mental health and center my attention specifically on how modalities of critique took shape in research, schools, clinics and everyday life (Fassin 2017). The story I recount begins not in the 2010s with the rise of GMH and the rapid proliferation of formal publications on adolescent sexual and mental health, but in the 1990s, the decade when young teens were first coming into contact with small scale initiatives in research, schools and clinics that fed this area of growing concern. It was during this time that young women were also encountering discourses and practices that sought to critique such initiatives. This “pre-historical” moment was comprised of a dynamic landscape of multiple and often conflicting knowledge-forms and values concerning young women’s passage to adulthood. How, I thus ask, did practices of critique take shape *prior* to the epistemic consolidation of the “adolescent sex-and-psyche” object of expertise?

The material I present draws from a historical ethnography that I have carried out since the mid-1990s in Pelotas, a medium size city in Southern Brazil that counts on a rich tradition of psychoanalysis, social psychiatry, social medicine, critical pedagogy and grass-roots mobilization. In addition to participant observation in a range of settings, I have conducted semi-structured interviews with over 100 individuals: psychiatrists, psychologists, social workers, educators, school directors, public health officials, politicians, researchers, activists working in non-governmental associations and religious leaders. In 1997, I was invited by researchers at the Department of Social Medicine (DSM) of the Federal University of Pelotas (UFPEL) to develop an ethnographic sub-study embedded in the 1982 Pelotas birth cohort study, a longitudinal epidemiological cohort of all children born in 1982 (Victora and Barros 2006).^4^ For this sub-study, we selected a group of 96 young people and visited them repeatedly from ages 15 (1997) to 25 (2007).^5^

In what follows, I begin by describing the incremental growth of clinical-pedagogic forms of researching and managing teen sexuality, pregnancy and psyche, and the problems this created for young women and their families. I take as a point of departure young women’s interactions with one particular technology of this management—the epidemiological questionnaire—and center specifically on  participant’s seemingly contradictory answers to questions regarding the intentionality of their pregnancies and the psychological upheaval that “early” sexuality and motherhood may or may not produce.^6^ I interpret these “contradictions” to be an intentional form of experimental epistemic micro-politics demonstrative of what Charles Briggs and Clara Mantini-Briggs have insightfully described as the search for “health/communicative justice” (Briggs and Mantini-Briggs 2016: 242). To situate this micro-politics, I use a wide-angle genealogical lens, tracing the ways histories of education reform, psychiatry, social medicine, critical epidemiology, reproductive health, the transition to democracy, feminism and grass-roots politics all entered the fold, shaping the ways young women, parents and professionals grappled with the contested rise of an “adolescent-sex-and-psyche” episteme (Dreyfus and Rabinow 1982).

I then shift to a granular analysis of the critical politics I observed unfolding. I show how in the same way young women sought to resist essentializing gendered and classist representations of the sexual and psychological life of low-income teen girls, so too did they struggle with the over-determined framings used by well-intentioned teachers, clinicians and researchers who sought to be sensitive to the forces of culture and poverty in young women’s lives. As scholars have shown, attempts to increase awareness of the cultural and social dimensions of health in clinics and public health programs often end up reifying long-standing structures of power (Duncan 2017; Fassin 2001; Metzl and Hansen 2014). I will show how young women, in responding to these structures, sought to tinker with the systems that produce knowledge and value, grappling with the histories and practices that delineate how health problems are defined, investigated and intervened upon, and perhaps most importantly, who gets to decide.

## Surveilling Sex and Psyche

It was late afternoon on a winter’s day of 1997. I was on my way home after a day of interviews in the *favela* –or *vila,* the term many residents prefer—when a group of girls I knew invited me into a young teen’s home to hang out and shelter from the cold. As my presence receded into the background, they began chatting about boyfriends and sex, disclosing intimacies usually reserved for circles of best friends. Marisa,^7^ then 16 years old, announced that her period was delayed. Her slight smile accompanied by the girls’ emotional gasps of surprise suggested that this was potentially good news. Over the course of the ensuing weeks, I learned more about Marisa’s good-news hopes. Her relationship with her boyfriend had intensified and her menstrual delay might end in a happy result: partnership, motherhood and fulfilling family life.

Marisa was not alone in her forthright embrace of motherhood, but like other young women, she generally disclosed her position only in private. In public, most young women and their parents ascribed to the ideal that teens should delay marriage and childbearing in favor of education (cf. Santos 2012). This ideal has been in the making for well over a century in much of Western Europe and the Americas through large-scale structural changes: the passing and implementation of child labor laws, the legally-mandated democratization of public education, growth in access to fertility control, trends favoring gender equity in education and employment and the professionalization of psychological and developmental expertise (Ben-Amos 1995). In Brazil, structural changes such as these, while also long in the making, intensified in the 1990s and 2000s. During these decades, an unprecedented proportion of the Brazilian population became upwardly mobile and primary school enrollment rates increased rapidly, reaching near-universal levels, especially in the South of the country (Santos Filho 1992). All-age fertility rates declined sharply, making families smaller, and reproductive health programs began targeting teens (Victora et al. 2011). Growing access to publically-funded mental health services situated in a network of decentralized primary-care clinics and public schools created a ready-made platform for psychiatrists to become increasingly involved in tending to young people’s psychosocial, pedagogic and sexual development (BRASIL 2007).

Despite these structural changes, the pedagogic and psychological institutionalization of child development is by no means universal across Brazil. As in other countries, wealthy and self-identified white youth are more likely to experience the protracted and well-orchestrated childhood and adolescence described in textbooks (Lesko 2001; Lima 2018). Further, young women’s bodies, sexualities and psyches have tended to become the prime focus of attention when educational and developmental ideals falter *because of*—so the clinical literature often assumes—a teen pregnancy (Heilborn et al. 2007). Growing emphasis on psychological risk factors for teen pregnancy—such as immaturity, sexual impulsivity, and inattention—and a key consequence of teen prengnacy—namely prolonged depression—has tended to shift the focus away from other forces such as access to education and social welfare (Koffman 2012). In Brazil certainly, the reproductive life of poor white and, in particular, non-white women has long been a target of pathologizing rhetoric, and the recent surge in alarmist discourses regarding teen pregnancy show considerable continuity with these historic threads.^8^

When I started my research in the late 1990s, I found young women and their families responding to the rise of psycho-pedagogic understandings of teen sexual life in a range of ways. I met several parents who actively sheltered their children from the institutional settings that they felt maligned their life-ways, refraining from “too much” contact with doctors and sometimes sending their children to school for primary education only. Sheltered modes of child-rearing were especially accentuated among rural migrants to the city, as was the case with Marisa’s family. For these families, teen pregnancy did not become the life-shattering experience that psycho-pedagogic discourses typically assume it to be. While few parents openly sanctioned premarital sex, it was broadly acceptable for young girls to become sexually active by around age 16/17, so long as *single motherhood*—the more problematic issue at hand—was avoided.^9^

Nevertheless, with the growing pathologization of teen pregnancy, the young women I met felt increasingly caught between two conflicting moral systems—one that stigmatized teen motherhood and another that celebrated it, if in increasingly veiled ways (Gonçalves and Gigante 2006). Marisa’s menstrual delay ended up being only that, but when she became pregnant a few months later, she was excited and began plans to set up a new home with her future husband. Like other young women, Marisa began responding to the feelings of shame and infantilization she experienced as an expectant mother, particularly in school, by actively associating with “traditional family values” and by subtly positing the moral superiority of her life choices against those of more “modern”—and in her view overly sexualized—peers. By the second trimester of pregnancy, Marisa had left school altogether.

In contrast to Marisa’s experience, a good many families I met living in the *vila* were more optimistic about the prospects of, as they put it, “modern life.” Parents noted that the possibility of completing secondary education and improving on household earnings, even significantly, no longer seemed as remote as it had once had. Those who had achieved some amount of upward mobility spoke about their children’s “development” with a clearer teleology in mind and they tended to problematize the simultaneous engagement in education and romance, particularly for girls.

I will never forget the look of trepidation and fear on Ana’s face when she told me, then 17 (1999), that she had just discovered she was pregnant. She had disclosed this to no one other than me and her older sister. “I was so worried about what my parents would say.” Ana explained, “My mother has always talked about how important it is for girls to remain in school so we can get a good [formal-sector] job with *cateira assinada* [benefits].” Ana’s mother was adamant about this; in her generation, it was not uncommon for women to have to ask their husbands for a stipend or for permission to work outside the home. But Ana was madly in love and she knew in her heart of hearts that her boyfriend would neither abandon her nor curtail her desire to remain in school. Even so, while her situation was nowhere near as difficult as some of the single mothers she knew, she felt scared and confused.

Approximately two years after became young mothers, Marisa and Ana participated in the 2001 survey of the Pelotas 1982 cohort study, conducted when participants were approximately 19 years of age. The study and its surveyors, generally medical students, sometimes became the topic of animated conversation, much in the same way gossip circulated when other “outsiders”—doctors, police officers, politicians—visited the *vila*. In the context of one of those conversations, it came to my attention that Ana had responded to a question about the intentionality of her pregnancy by stating it had been planned.^10^ I found this curious. From everything I had known of Ana’s life, this was in fact not the case. In contrast, Marisa, who also volunteered her responses, stated her pregnancy had been “unintentional.” She had indeed not set out to have sex for the express purposes of getting pregnant, but compared to Ana, her pregnancy had seemed to me far more intentional, a sort of accidental happening that she and her family welcomed.^11^

The 2001 questionnaire also included questions about mental distress. In line with the principles of psychiatric reform and social medicine that prevailed at the time, the researchers avoided adopting specific diagnostic categories from the International Classification of Diseases and instead used a generalized morbidity screening tool, the SRQ-20. They also measured the social and economic determinants of health and asked about stressful life events and “nerves.”^12^ Here again, I found Ana and Marisa’s answers curious when contrasted with what I had learned about their emotional lives. As Ana transitioned to motherhood, settling into new family relations while struggling to remain in school, she spoke vividly about her *nerves attacks* and yet on the questionnaire, she claimed to have never had any *nerves* problems and scored high on the SRQ-20. Marisa’s newfound relations with her in-laws also caused her persistent *nerves* problems, yet she scored low on the SRQ and claimed on the questionnaire to have never suffered from nerves.

What was the significance of the disjuncture between these young women’s data-statements and what I (thought I) knew of their life experiences? Was this an example of technocratic reductionism in the making or was there something more intentional in the profile young women were registering? In what follows I will argue that young women were using the questionnaire as a semi-deliberate tool of critical micro-politics. Before demonstrating this, I will first sketch the multiple genealogical threads that were, in coming together, opening space for a materially-grounded, structural and praxis-oriented form of experimental critique.

## Genealogies Colliding

When I met Zilda in the mid 1990s she was working as senior pedagogic advisor and director of the *Serviço de Orientação ao Educando* (SOE), the “Student Services Office” in a large public school. Like in many schools, the SOE was staffed by a psychologist and learning specialist. “I always tell young people,” Zilda responded when I asked about teen sexual development, “there is a big difference between being biologically prepared for reproduction and having the psychological maturity needed to become a parent. These kids don’t know if they are children or adolescents and this leads to developmental agitation, *indisciplina* (lack of discipline)… school failure, aggression, sexual risk-taking, and even drug and alcohol abuse.” Zilda’s interest in understanding how psychosexual development linked to broader behavioral problems was becoming more routine at the time. I interviewed teachers, psychologists and pedagogic specialists who reiterated what one school director said quite succinctly: “Young girls struggling in school and exhibiting agitated behaviors… are predisposed to engaging in sexually demonstrative behaviors and early sexual initiation.” The core idea here was that living in poverty “exacerbated” underlying developmental agitations. For instance, Andrea, a teacher, told me that teen childbearing was more likely among “alienated youth” from the *vila* who had poor “impulse control.” Though “poverty” framed such discussions explicitly, indirect references to how black and mixed-race youth seemed particularly prone to sexual precociousness often brewed under the surface.^13^

It was around this time that teachers began referring students to the school psychologist not only for “inattention” but also for a growing number of “co-morbid” conditions, including “sexual impulsivity” or “sexual precociousness.” At times, pediatricians, obstetricians and psychologists—more so than psychiatrists or public health experts—were called upon by school directors to provide assistance with problem students, with solutions often consisting of educating teachers on the principles of young people’s sexual, emotional and cognitive development. Research on the health risks produced by the so-called temporal widening of the interval from sexual to psychological maturation also began to be conducted.^14^ The shift to an interest in development and maturation helped justify the need for earlier psycho-pedagogic interventions for problems surrounding sexual behavior in addition to learning.^15^

A range of critical voices came to the fore. Psychiatrists committed to community mental health were concerned that young women being referred to their clinics from “problem schools” were arriving with a ready-made language of immutable behavioral pathology. One leading psychiatrist in the city explained, “Those in the school can be very quick to tell us, ‘oh this child has inattention,’ but the school can be a very chaotic environment…. And sometimes there is a lot going on at home… the family just needs some support.” According to some of my interlocutors, the psychologization of teen sexuality was more common in large peri-urban public schools where school directors reported intense teacher-to-student and peer-to-peer conflicts. Therapists worried that they were witnessing the beginnings of an “impending wave of over-medicalized” and pharmaceuticalized responses, akin to what had started happening in the US in the 1980s. Of all forms of medicalization, those centered on teen sexuality seemed to some of my interlocutor-critics the most problematic.

It is important to underscore just how much was at stake in these kinds of critiques. The reforms of the 1990s and 2000s rekindled long-standing debates relating to Brazil’s history of nineteenth-century hospital-based psychiatry and early twentieth-century importation of psychoanalytic texts (Costa 1976). Historians influenced by Foucauldian thinking wrote critical histories of Brazilian psychoanalytic psychiatry with some arguing that “old-school” conservative interpretations of Freud’s works had not been as radically critiqued or deconstructed as some had claimed.^16^ Anthropologists showed how psychoanalytic ideas and techniques continued to function as they long had—as a core civilizing technology used by the elite to justify the economic and racial marginalization of those deemed too “intellectually” rudimentary for the work of introspective analysis (Duarte 1999–2000). Throughout psychiatric reform, however, psychoanalysis was not thrown out with the bathwater, in part because the critique of psychoanalysis went hand in hand with the kind of reflexive genealogical work to which those leading reform were committed (Russo 2014).

Critical perspectives also gained traction in faculties of social medicine where professors began to argue that mental health was a problem of society rather than individual psyche. Epidemiologists at the UFPEL, for instance, began pushing for more attention to the social determinants of young people’s health, showing in particular that teen pregnancy rates were higher among youth with minimal levels of education and from families with low income of low-income, as well as among those who self-identified as having “black” or “brown” skin-color (Gigante et al. 2008). Community doctors also worried that the rise of adolescent mental health expertise was little more than a strategic market-driven fad. “Adolescents don’t really get sick [or] die in great numbers,” explained one such doctor, “so really it becomes an issue of teen pregnancy, violence, drug use, school problems…but are these clinical or psychological problems? Will we resolve them with adolescent medicine or adolescent psychiatry?” It was around this time also that HIV-AIDS/STD prevention campaigns, particularly those geared towards youth, began intensifying across Brazil. In Pelotas, some of these initiatives were designed by public health specialists who had no particular interest in psychologizing adolescent behavior. In fact, some were clearly opposed to such a move. Celia, a reproductive health researcher who worked closely with the local government conducting workshops in schools told me that high fertility rates, whether for adults or teens, had less to do with sexual impulsivity or contraceptive knowledge than gender inequity. Like others who promoted gender empowerment, Celia’s approach was informed by her reflections of the coercive reproductive campaigns and human rights abuses that have taken place at various points in Brazilian history under the umbrella of “population control” (cf. de la Dehesa 2019).

Debates within education also began to surface more forcefully. A handful of school directors explained that the growth of a medical-developmental language centering on risk-behaviors was in fact an enduring trend that had started intensifying during the dictatorship. It was well known that the regime had opened a window in the 1970s for the dissemination of behavioral theorists (such as B.F. Skinner from the US) in schools where keeping order was tantamount (Barbosa 2012). Vilma, a pedagogic advisor, explained, “At the time of the military, psychology already played an important role in schools, but it was of a different sort. Psychologists were there to be moderators, to appease and calm everyone so that the system could function and so that the conflicts underneath would not emerge.” With the return to democracy, those leading education reform set out to tackle pacifying uses of psychology as well as old-style teaching methods centered on rote memorization and authoritarian forms of discipline, shifting towards child-centric “democratic” philosophies (Gadotti 1992).

Yet such initiatives were fraught and everyday conflict seemed ready to implode. In some schools, students gained a voice through their election to newly-formed student councils and they began organizing for students’ rights, but teachers complained that the distinction between “empowerment” and “misbehavior” was tenuous. One teacher, Julia, told me that her students from the poorest *vilas* were the most volatile and hardest to teach and that maintaining discipline had become exceedingly difficult because the “boundaries between school and *favela* were becoming too porous.” To keep order, school staff often described falling back into “old-school” teaching styles. Students rebelled, refused to participate and were increasingly referred by exasperated teachers to the school psychologist. Even so, those leading reform persisted—and sometimes succeeded—in reshaping the place of psychology in schools, in part by following the ideas of critical pedagogic theorists such as Paulo Freire, encouraging teachers to adopt pedagogic styles that fostered not conformity but what Freire famously termed “critical consciousness” (Freire 2005 [1970]).^17^

Most young people did not find the critical discourses that were circulating in schools and clinics compelling, in large part because intense levels of disillusionment and conflict have long characterized families’ interactions with a whole host of authoritative institutions (Scheper-Hughes 2004). But a not-so-insignificant number, Ana included, remained engaged in school, at times arguing with her teachers, at other times participating in class and soaking in the ideas of critical pedagogy, however incipient. It was no coincidence that these young women tended to have parents or extended family members who had been politically active at the grass-roots level in the lead-up to the ousting of Brazil’s dictatorship and who continued participating in their neighborhood organizations, regularly debating political issues and lobbying the local government for residents’ rights (cf. Holston 2009).

## A Saturated Tool of Politics

In the same way that some families gravitated towards small-scale activism, shaping and drawing inspiration from some of the critical genealogical I have just traced, so too did their daughters come to view the epidemiological survey not as a mere register of confidential information but as a way to “speak to” elite authority—that is, as a semi-public tool of communicative health/justice (Briggs and Mantini-Briggs 2016). The survey, to recall, was administered face-to-face, usually by a middle-class university student in the young person’s home. Compared to other usually more suspect “outsiders” who sometimes came to the *vila*—politicians, the police, government official, or even the community doctor—the survey interaction stood out, for no matter how constrained by its multiple choice questions, it was both an act of listening and a technology that revealed the ways of reasoning through which *vila* populations are managed and doctored. Though Ana appreciated the confidentiality the cohort study ensured, she voiced curiosity about the final report and what she might convey therein, and once asked me directly if I knew what it would conclude.

What then did Ana’s “intended pregnancy” as registered on the questionnaire mean? In most direct terms, Ana’s desire to tick the “intended pregnancy” box was part of her broader attempt to counter normative pedagogic, public health and psychologized framings of teen sex and psyche. “I knew exactly what I was doing,” Ana said, pinpointing the precise week during which she had had unprotected sex. Similarly, I spoke to other women who explained that “lack of knowledge about contraception” and “being irrational in the heat of the moment”—the key explanations they said “teachers and doctors” referenced for teen pregnancies—were incorrect. Dienifer, for instance, said, “Sure, I was going through a rebellious phase around the time I became pregnant, I got a reputation … I wasn’t sure my boyfriend would own up, but you can’t say I didn’t know what I was doing…. I have no regrets.” Of the 10 women from the ethnographic sub-study of the cohort who became mothers before the age of 20, five registered their pregnancies as having been intended, even though from what I learned of their lives at the time, these were in fact accidental events that were then reconstructed as partially-planned.

Fueling these young women’s desires to assert the legitimacy of their motherhood was a dual sense of attraction to and dissatisfaction with the promises of what they referred to as “modern life.” Countering wide-spread representations of unsuccessful students as either “lazy” or “unintelligent,” some young women were quite forthright about how unsupported they felt in schools. For Rita, as for others, pre-emptive interventions in schools—referrals to psychologists, sex education classes, teachers’ subtle warnings to not date “too many boys”—compounded their ongoing sense of intense social exclusion. As Ana told me, “What those [psychologists] say, is just *cheio de frescura* (pretentiousness) [….] As soon as I hooked up with Marcio, they kept saying that I didn’t seem interested in my studies anymore. But all girls date!” Dienifer described the many ways that constant tensions surrounding the behaviors of young people from the *vila* “end up leaving you all *marcado* (marked up),” a term widely used to refer to students who acquire a “bad reputation.” Another young woman similarly explained, repeating the motto-like statement, “if you are not progressing in school, they say it must mean that you are making babies [having sex] at home.”

While the depictions women encountered were often infused with racialized undertones, young women’s responses, regardless of how they identified in terms of race, skin color, or ethnicity were virtually always articulated through a politics of class. The more young women were exposed to the notion that their sexualities and psyches were underpinned not by romance and budding adulthood but by developmental immaturity compounded by life in poverty, the more they defiantly affirmed their identities as proud young mothers. A key source for this defiance, beyond families and neighborhood- or street-based activism, was the school itself. In fact, Ana and others stayed in school in part because they wanted to become politically active in a space that was more intimately visible to middle-class authority. Several, for instance, recounted with some enthusiasm how they had participated in petitions to improve school conditions circulated by their student representatives.

Modes of signification on the questionnaire were also linked to attempts to counter gender normativity. To recall, young women like Ana had been explicitly reared to delay motherhood in favor of the potential emancipation that completing secondary education might provide. Sometimes, this form of child-rearing took place at the displeasure of young women’s fathers and with considerable marital struggle. For some fathers, a prolonged adolescence via extended education typically meant too much time for dating, which put young women in a risky situation because they might lose their virginity with someone other than their future husbands and/or acquire a “bad” reputation.^18^ Young women were sometimes secretly supported by their mothers to stand up to their fathers, and in so doing sometimes found an ally in those leading school-based reproductive health workshops that centered on gender equity. So divisive were prevailing values relating to teen sexuality that I observed friendships split over them: while “emancipated” young women like Ana and Dienifer were often denigrated by peers for being “morally loose” or even “snobby” in their upwardly mobile ambitions, women like Marisa were criticized for being stuck in “traditional” values and “acquiescent” feminine ways. Thus, when Ana and Dienifer registered their pregnancies as intentional, they were also rejecting maligning views of their sexuality and asserting the moral suitability of their feminist inclinations.

Marisa’s relative absence on the questionnaire was a clear counterpoint to Ana’s positionality. She, like others, said her pregnancy had been unintended even though motherhood had unfolded as a largely expected and celebrated event. In time, I came to understand that Marisa was simply not vested in the survey itself. Somewhat like school, she viewed the research encounter as a skewed apparatus used by a distanced and powerful upper-class. Eloisa’s position was similar: “I don’t need to justify anything to anyone. I know people are against teen pregnancy, but for me, it was the best thing that could have happened. You also learn a lot being a mother …. What do I want with ppt [school, also known as *preparacao para trabalho*—preparation for work] anyway? In reality [school] is ppn [*preparacao para nada*—preparation for nothing].” Eliosa and Marisa were quite determined in their affirmations as morally upstanding mothers and wives, and they identified as anything but “teen mothers.” Fernanda, for instance, who became a mother at age 18, once clearly distanced herself from the moral impropriety of “real” teen pregnancy by stating, “Things are so different today, you see 12 year-olds going to the hospital to give birth; children having children! Like they say, at the time [of having sex], they didn’t act like children.” Though these women were opinionated in private, their responses on the questionnaire were not crafted for an outside audience. To state the pregnancy had been unintended conformed with what they knew the interviewer expected to hear and it aligned with their desire to simply disengage from the educational and clinical contexts that judged them.

In contrast, Ana’s outward-facing politics did not stop at the question of the significance of her pregnancy. Elsewhere, I have described how young women’s impetus to resist gendered discourses gained momentum alongside their desire to use the very behaviors that were becoming objects of concern to assert their “right to do the same things as boys” (Béhague 2018). Often going against their parents’ wishes, Rita, Ana and Dienifer went to parties and nightclubs; they hung out on the street corner with friends instead of at home; they acted up in school and were frequently sent out of the classroom; and when they were referred to school psychologists, they neither acquiesced to nor ignored the psychologist’s urgings, as most young people did. “Sure it’s good to have someone [a therapist] with whom you can *desabafar* (unload),” said Rita, “but I went and she [school psychologist] didn’t do anything. She just asked questions. She didn’t explain why the teacher was picking on me and not the others. I told her this.” Both Rita and Ana began to show subtle pride in the prototypically “masculine” psychological languages centered on “conduct” and “agitation” that had begun to form around them. I was only partially surprised to find that when asked about “risk behaviors” in the questionnaire, the five young women in my ethnographic study who had classified their pregnancies as having been intentional also answered that they smoked regularly, had gotten drunk several times and had dated dozens of boys. While they did indeed have more freedoms than most, from what I had observed of their lives, these were clear exaggerations.

I found myself going back to the larger epidemiological database, thinking of statistical associations as embodiments of social patterns that young women actively fiddled with. It turns out that the 25% of women who stated on the questionnaire that they had intended on becoming a teen mother relayed behavioral patterns typically associated with adolescent boys (partying, smoking, drinking, dating) in a greater proportion than the 75% who stated their pregnancies had been unintentional. These same women also identified with a particular kind of diagnostic language in the questionnaire. Though the cohort researchers refrained from using specific diagnostic tools, they did ask young people if they had ever visited a doctor, psychologist or psychiatrist for an emotional difficulty; if the answer was yes, participants were asked to describe the motive for care in their own words. Young women who had visited a clinician for behavioral problems prototypically associated with boys (inattention, conduct problems, learning difficulties, agitation) rather than those typically associated with girls (nerves, anxieties, traumas, difficult life events) were also more likely to have registered an intentional pregnancy (Béhague n.d.). In mainstream psychiatric epidemiology, this clustering is generally interpreted to indicate co-morbidity and an underlying disorder. From all I had learned, however, the profile young women were registering on the questionnaire, which I took to be partial truths and partial overstatements, reflected the desire for a semi-public form of intersectional class/gender politics.

## Ontological-Historical Experiments

Young women’s answers to the questionnaire were part and parcel of a larger form of personal politics that reached well beyond questions of representation. That is, they approached the questionnaire as they did the school, family-life, street-life and the clinic: as a techno-experimental space for invigorating an ontology of micro-political action that took shape at the intersections of gender, class, psyche, adolescence, kinship and more. Allow me to explain:

The descriptors Ana, Rita and Dienifer selected on the questionnaire served as proof that they had not meekly turned away from schooling and “modern life;” that their struggles with middle-class authority were fraught and emotionally exhausting. When they spoke of their anxieties and *nerves attacks*, it nearly always included a discussion of the judgments that others—and, in particular, middle-class others—held of them, first as resident of the *vila* and second as “poor teen mothers.” More so than an outgrowth of the invariable challenges of motherhood itself or life in poverty, young women’s emotional upheavals were, as they themselves explained, linked to the fact that they “came forward and fought,” exposed themselves to discrimination and did not fade away into the world of “tradition,” as did, they told me, some of their childhood friends.

Though this fading away was the position Marisa adopted, in my view, her form of politics was not passive or “traditional” but centered on protection from the damaging depictions of her motherhood as being rooted in—and the cause of—psychological problems. As colleagues and I have shown elsewhere using ethnographic and epidemiological analyses from the cohort, the association between young motherhood and mental distress held only for those women who on the questionnaire relayed having experienced economic or racial discrimination, and who sought to counter this with political action (Béhague et al. 2012). In my interpretation, this particular form of suffered young motherhood signals the making of what Margaret Lock has termed a “situated biology,” a contingent entanglement of the biological and social (Lock 2013).

To underscore this entanglement, Ana, Rita and Dienifer *wanted* to use the language of authoritative institutions and they knew the SRQ-20 was that language. It was for this reason that they under-emphasized their *nerves attacks* on the questionnaire. Ana once told me that “*nerves* were seen as backward,” even by doctors who asked specifically about them. In the absence of actual changes in daily relations of othering and discrimination, references to nerves, particularly when made by a member of the elite, just cast her deeper into marginality. As Duncan has shown in her study of psy-initiatives in Mexico, “culture bears the brunt of critique [not because experts] are unaware of the cultural, social and structural determinants of mental illness but because conceptual opposites are built into the very enterprise of modernization” (Duncan 2017:46). It was not that Ana considered nerves unimportant or phenomenologically the same as that which the SRQ-20 described, but she sought a mode of politics that might unsettle these conceptual opposites.

In this regard, it is important to underscore that young women like Ana, Dienifer and Eliete were doing more than *resisting* widespread gendered and classist stereotypes of low-income teens. Their survey answers reflected the ways they actively sought to scramble expert knowledge and *rewrite* the future that preemptive scientific knowledge of teen-hood and psychological risk foretells. To use Briggs’ and Mantini-Briggs’ phrase, they were engaging in “conceptual border crossings” into the world of epidemiology in an effort to unsettle the powerful co-production of knowledge and the subjects of that knowledge (Briggs and Mantini-Briggs 2016: 242). By remaining in school, registering their prideful intentional pregnancies, taking on prototypically masculine behaviors, and exposing themselves to institutionalized values and practices that constrain and wound, they embodied mixed epidemiological profiles that defied normative patterns embedded in statistical associations. In so doing, they experimented with inhabiting a new ontological-affective space. What united these disruptive efforts was the practicing of a both/and mode of being amidst either/or structures and values that posit family or education, poverty or upward mobility, *vila* life or middle-class existence as irreconcilable opposites. Ana was becoming neither a traditional home-bound wife nor an immature and impulsive teen whose pregnancy could somehow be interpreted as on par with drinking or drug use: she was in other words incrementally overturning “the stylized repetition of acts … that make gender” (Butler 1988:522).

At times, young women’s desire for this kind of experimentation melded into community mental health practices that were taking shape at the time. Here, genealogical threads emerging from psychiatric reform and social medicine came bubbling to the surface more forcefully. Recall that in her earlier teen years, Ana had somewhat atypically showed up at the school psychologist’s clinic, curious and with her own agenda. This curiosity persisted. Later in her early 20s, she sought a psychiatrist, this time via her local primary care clinic. Here again, she had no interest in talking about her nerves, nor even the emotionally debilitating effects of her precarious life. These topics she reserved for friends and family. Instead, she arrived ready for a struggle, initiating clinical talk by returning to the diagnostic language of adolescent impulsivity and sexual impropriety that had swirled around her behaviors in years prior.

Using what Douglas Holmes and George Marcus have termed “para-ethnographic” modes of reasoning (Holmes and Marcus 2008)—together with what one might also call “para-epidemiological” understandings of how psychological languages are differentially distributed in society according to gender and class—Ana began picking normative knowledge apart, outlining a radically different interpretation of the agitations, nerves and impulsivities she inhabited. She told the psychiatrist in no uncertain terms that social conflict, judgments and *preconceitos* (prejudice) were central to the depressions and anxieties she felt. Similar therapeutic trajectories took place with Dienifer, Eliete and others, even when clinical encounters were quite short-lived. To the clinic, they brought stories of conflict—allegations that teachers unfairly targeted students from the* vila*; confusing feelings of anger when being overly-scrutinized by middle-class peers; stories of interactions with adults who held dramatically distinct views on whether their gender contestations signified adolescent pathology or a new politics of feminist emancipation.

History also came to matter very much in these conversations: histories of family members’ experiences with classist accusations in places of employment; histories of mothers’ psychiatric hospitalization often in situations of duress and suspect forms of care. That some young people sought to discuss these issues with not just anyone but with a therapist whom they viewed as a representative of the elite was highly significant. As I have shown elsewhere, what ensued was productive in part because of therapists’ willingness to make of the clinical encounter a space of social conflict, of the rendering accountable of those in power, and through this, of addressing young women’s moral and political worthiness (Béhague 2009).

## What Kind of Critique

What then can we learn from this case about practices of critique? Let me first hypothesize that modalities of critical engagement might be distinct before the consolidation of an object of expertise and its defining features. In the case of psychiatry and GMH, these defining features tend to include specific diagnostic categories, theories of disease causation that center on “down-stream” determinants, and cost-effective models for identifying treatments. We might further hypothesize that the formalization of these features also delineates, to some extent, the shape of critique. This shape, I wish to argue, is one that tends towards a politics of epistemic struggle underpinned by a hermeneutics of suspicion. Rita Felski has written that “suspicious” critique is more than an intellectual or conceptual intervention; it is an ethos and aesthetic, a mood that unfolds in contingent moments and with particular effects that should be studied as real-world phenomena (Felski 2015). This aesthetic, centrally present in medicalization theory, informs many critiques of GMH. Descriptions of “hegemonies of” and “resistances” to GMH rest on the assumption that systems of power are best disrupted by deconstructing the defining features of expert knowledge-systems noted above. I do not wish to claim that this kind of critique is not useful and important, but it is arguably only one of many possible forms (Fassin 2017).

The Brazilian “pre-historical” moment I have described can point us toward other complementary forms of critical practice. The kind of critique I saw at play was less fixed, more porous and only partially about psychiatric and pedagogic knowledge-production; it was experimental and grounded in practices that emerged from everyday material experiences and moral struggles. It is true that Ana was using elements of medicalization theory, indirectly arguing that interventions focused on the close management of reproductive and mental life were misplaced, particularly even offensive, when decontextualized from the social, cultural, moral, structural, historical forces that so visibly disenfranchise. But she did not respond by asking others to bear witness to her culture or precarious life, nor did she allow herself to become emblematic of that culture or precarity. At the forefront of her struggles was not cultural translation or diagnostic contestation. Though these struggles were also important, what Ana and others seemed to most desire—and what they were at times encouraged to seek—was agency to enter into, create and uphold new structures that redress deep inequities in how distinct forms of knowledge about mental life are distributed, used and valued.

As an emerging “co-expert,” Ana’s theorizations went beyond resisting epistemic conventions in psychiatry, for she sought to redraw core hierarchies of social power (Rose and Kalathil 2019). Her experiences remind us that appealing to cultural and structural dimensions of health can, as with the biological, become powerfully over-determining and essentializing. The contingency of this reductionism was something researchers and clinicians grappled with, and at times, what mitigated over-determination, whether it be biological, social, cultural or any other “-al”, was ensuring people be given the worth, value and means to analyze and re-make their world. As Joao Biehl and Adriana Petryna have written, “People constantly exceed the projections of experts. The medicoscientific, political and humanitarian frameworks in which they are temporarily cast cannot contain them. […] We must hold social theory accountable…” (Biehl and Petryna 2013: 5).

Arguably, the growth of audit-oriented and cost-effective models in global health pressure assemblages such as GMH to account to donors and agencies more readily than the people they are meant to assist (Adams 2013). But it is also useful to ask what it might mean for critique to remain accountable to people and their everyday realities. Ana’s mode of critical engagement for instance underscored the importance of both *analyzing* enduring forces of dispossession and *opening* a space for non-prescriptive and non-formulaic alternatives that might in the future blossom. This requires experimenting at the edge and limits of theory. Paulo Freire has taught us that critical consciousness is not only about the development of specific concepts that render the ‘social’ visible. Critical consciousness depends on iterative processes between action and reflection: “reading the world,” he wrote, is contingent upon also “rewriting it” (Freire 2005 [1970]). I have too little space to delve into the inspiring anthropological works that, in my reading, share in this Freirean aesthetic, one that takes a hermeneutics of suspicion not as formula but point of departure: the possibilities of an *otherwise* (Povinelli 2012), the inspirations of design theory for new world-makings (Escobar 2018), and the anthropology of becoming (Biehl and Locke 2017) are just a few.

GMH proper is too new in Brazil to be able to clearly identify either how it might crystallize or whether Brazil’s still somewhat diverse landscape might contribute to decentering normative trends in GMH. Some of the genealogical threads I have described—from critical pedagogy, social psychiatry, feminist reproductive justice, epidemiology of inequity, and grass-roots movements, to name a few—are increasingly under threat in Brazil and may or may not end up being constitutive of how the GMH assemblage takes shape. It is certainly worth trying to not throw the babies out with the bathwater. The most recent Lancet Commission on GMH, situated in the much broader framing of the Sustainable Development Goals, mentions “social interventions” more than any other formal publication to date (Patel et al. 2018). It remains to be seen however what kind of “social” might gain traction and if the conventional emphasis on individual-level cost-effective treatments will remain par for the course.

Nikolas Rose has argued that another biopolitical role for psychiatry *is* possible but will require more than cultural sensitivity or a rights-based approach; it will need “a reformatting of the social control functions of psychiatric expertise” which includes, amongst other shifts, “the recognition that, if [psychiatry] claims to be for the benefit of those who are its subjects, those subjects… must have the leading role in judging its successes and failures” (Rose 2018:193–195). To open up such a space, however, we also need to study critique itself in order to learn *how* this “reformatting” might unfold. Only then will we begin to understand how the productive epistemic multiplicities that have been brewing in the Brazilian landscape might feed into a more flexible and responsive global mental health.

## Footnotes

1 For an in-depth analysis of the epistemic positions and practices associated with GMH, see Bemme and D’souza (2014), Kirmayer and Pedersen (2014) and Lovell et al. (2019).

2 For instance, two of the co-authors of the 2011 Lancet article cited above are Brazilian psychiatrists situated in departments known for being highly productive centers of biomedical psychiatry.

3 Being a so-called middle-income country with a national health care system and long-standing schools of medicine, psychiatry and psychology, Brazil was not considered a high priority country for GMH assistance but nor has it contributed in any significant way to GMH knowledge-production.

4 Ethics clearance was obtained from the UFPEL. Participants provided written consent at the time of the epidemiological surveys and verbal consent for the ethnographic sub-study.

5 Fieldwork, comprised of participant observation and repeated semi-structured and informal interviewing with youth and their families, was conducted by myself and a team of five ethnographers. The 96 young people were selected randomly, not to conduct probabilistic analyses, but to capture an array of life experiences, including those of particularly marginalized, isolated and introverted youth.

6 Of the 96 youth in the ethnographic cohort, 45 were girls and of these 10 became teen mothers. These young women introduced me to another 15 teen mothers whom I also came to know well, though not for the same duration of time.

7 To protect informants’ identities, all names used in this paper are pseudonyms and some identifying details have been altered.

8 As historians have shown, the genealogical undercurrents at play in pathologizing framings of pregnancy among poor women hark back to mid-nineteenth and early twentieth century eugenics-oriented population control platforms underpinned as these were by theories of degeneracy (Carrara 2015).

9 In urban centers where state-funded health care has been fully decentralized and is generally well-stocked, young women have relatively good access to contraceptives and more knowledge of reproduction than is often assumed by public health researchers. While abortion is illegal, wide-spread black-market use of misoprostol has increased women’s access to termination (Rocha et al. 2007).

10 Several of the young women I write about in this article spontaneously volunteered the information they had provided in the epidemiological survey. All were also aware, as per the study’s informed consent sheet, that the ethnographic study was a sub-study of the larger 1982 birth cohort and therefore that the information they had provided to the epidemiological surveyors was available to the birth cohort team of researchers, myself included. However, I never asked women about the specific responses they provided on the questionnaire or why sometimes the responses they volunteered seemed at odds with what they told me ethnographic conversations; this information was either volunteered or inferred at a later date in the analysis.

11 Quantitative estimates of unintended pregnancies—which in the Pelotas 2001 survey stood at around 75% of the sample—are likely to be inflated in places where the standard presumption is that teen pregnancies are unintended and where the explicit desire to become a young mother is widely stigmatized.

12 See Duarte (1986) for an in-depth analysis of *nervos*, a so-called “indigenous” category of mental distress found in much of Latin America.

13 References to race or skin color rarely entered explicitly into professionals’ discussions and debates. This is both because of the active suppression of racial politics and Brazil’s long-standing myth of “racial democracy” (Goldstein 2003). Until relatively recently in the social sciences, the dominance of class as the main way of conceptualizing subjugation has dampened the creation of an analytical and political language for interrogating questions of race (Htun 2004).

14 Much of this was based on a postulate known as the temporal gap theory that was first proposed in the 1950 s by US developmental pediatricians, most notably James Tanner (Vitalle et al. 2003).

15 A handful of local primary health care clinics where health education discussion groups for pregnant women had long been in place began working more intensely with teens, preparing them for motherhood while also addressing the psychological dimensions of their now truncated “adolescences.” Some of those running these groups began to view pregnant teens as “carriers” of preventive public health messages for other difficult-to-reach teens.

16 For a cogent review of the theoretical shifts in this phase of the historiography as well as how these analyses influenced psychiatric practice, see (Facchinetti and Rafael 2015).

17 For Freire, “critical consciousness” is the process of gaining awareness about personal and social problems through action. It is fostered via open multidirectional dialogue and reflection, but crucially moves beyond this by stipulating that critical reflection depends on the ability to intervene in the world in order to change it (Freire 2005 [1970]). In what I observed, such approaches often meant turning the spot-light on teachers and subtle dynamics of discrimination in the classroom. For instance, a leading researcher at the Department of Education at the UFPEL wrote a series of influential papers on how teachers’ sensitivity to poverty often led them to lower standards for their students from the* vila* (Damiani 1998).

18 A related point of disagreement pertained to whether or not to allow young women the freedom to attend parties without a male chaperone, with some fathers preferring their daughters have a boyfriend both to protect their daughters’ safety and ensure gender-appropriate behavior.
